# Chemical Composition and Validation of the Ethnopharmacological Reported Antimicrobial Activity of the Body Fat of *Phrynops geoffroanus* Used in Traditional Medicine

**DOI:** 10.1155/2013/715040

**Published:** 2013-11-05

**Authors:** Diógenes de Queiroz Dias, Mario Eduardo Santos Cabral, Débora Lima Sales, Olga Paiva Oliveira, João Antonio de Araujo Filho, Diego Alves Teles, José Guilherme Gonçalves de Sousa, Henrique Douglas Melo Coutinho, José Galberto Martins da Costa, Marta Regina Kerntopf, Rômulo Romeu da Nóbrega Alves, Waltécio de Oliveira Almeida

**Affiliations:** ^1^Programa de Pós-Graduação em Bioprospecção Molecular, Universidade Regional do Cariri, 63105-000 Crato, CE, Brazil; ^2^Programa de Pós-Graduação em Biotecnologia de Recursos Naturais, BioMol-Lab, Departamento de Bioquímica e Biologia Molecular, P.O. Box 6043, Universidade Federal do Ceará, Campus do Pici, 60455-970 Fortaleza, CE, Brazil; ^3^Programa de Pós-Graduação em Etnobiologia e Conservação da Natureza, Universidade Federal Rural de Pernambuco—UFRPE, 52171-900 Dois Irmãos, Recife, PE, Brazil; ^4^Departamento de Química Biológica, Universidade Regional do Cariri, 63105-000 Crato, CE, Brazil; ^5^Departamento de Biologia, Universidade Estadual da Paraíba, 58429-500 Campina Grande, PB, Brazil

## Abstract

*Background*. *Phrynops geoffroanus* is a small turtle that inhabits lakes, rivers, and streams throughout South America. The body fat of this animal is used as a folk medicine in Brazil for treating illnesses such as sore throats, ear aches, mumps, rheumatism, and arthritis. The present study evaluated the antimicrobial activity of oil extracted from *Phrynops geoffroanus* (OPG), determined its chemical composition, and discussed the implications of its use in traditional medicine. The OPG was obtained from the ventral region of this turtle using hexane as a solvent. The antimicrobial activity of OPG was tested against standard and multiresistance strains of bacteria and fungi and its composition was determined indirectly by analyzing the methyl esters of the component fatty acids. The OPG presented a clinically relevant antifungal activity against *Candida krusei* ATCC 6258 (MIC 128 *µ*g/mL). When the OPG was associated with the antibacterial and antifungal drugs, was observed a synergistic effect when associated the OPG with the gentamicin against the strain *Pseudomonas aeruginosa* 22. Our results indicated that OPG has clinically relevant antifungal activity against *C. krusei*, and demonstrated synergetic antibacterial activity in combination with commercial antibiotics against *Pseudomonas aeruginosa.*

## 1. Background

Reptiles are frequently used in making traditional medicines and their roles in folk practices for curing and/or preventing illnesses have been recorded in many different social-cultural contexts throughout the world [1–4]. Historical documents show that animals have been used in traditional medicinal practices at least since the first colonization of Brazil [5], and studies by Alves et al. [6] have documented the use of natural products derived from reptiles (including leather, teeth, fat, meat, and bones) as foods and for ornamental and medicinal purposes in rural and urban areas of that country. Freshwater turtles are included among the many animals used in traditional folk medicine and their shells, blood, eggs, and body fat are sought after as zootherapeutic elements in northeastern Brazil [7, 8]. 

Among the 278 species of turtles in the world, approximate 20% occur in South America in 20 families; the most species rich family is Chelidae, with a total of 23 species (19 of which occur in Brazil, where they are popularly known as “cágados” [9].

*Phrynops geoffroanus* is a small turtle with a predominantly carnivorous diet [10]. It is popularly known as “cágado-de-barbela” and is widely distributed in South American countries [11, 12] in lakes, rivers, and streams with relatively large volumes of water [13].

*P. geoffroanus* is used by many traditional communities in Brazil as a zootherapeutic [14, 15]—principally its fixed oil from body fat—to treat illnesses such as sore throats, ear aches, mumps, rheumatism, and arthritis [16]. Many of these maladies (inflammations and dermatitis) are associated with pathogenic organisms, including bacteria and fungi, which suggest the existence of antimicrobial activity associated with this turtle species, although no laboratory studies have yet examined the efficiency of this popular folk remedy.

As such, the present work identified the chemical components of the body fat of *Phrynops geoffroanus* and evaluated its antimicrobial activity (either alone or in association with antibiotics and antifungal drugs) and discusses the implications of its use as a traditional remedy.

## 2. Methods

### 2.1. Zoological Material

Specimens of *Phrynops geoffroanus* were collected in the municipality of Aiuaba (06°36′ S × 40°07′ W) in Ceará State, Brazil, in September/2011 using active collection techniques [17]. The specimens were subsequently anesthetized using ketamine (60 mg/Kg) and xylazine (6 mg/Kg) [18] and sacrificed, and their body fat was removed. Reference specimens were fixed in 70% ethyl alcohol and deposited in the zoological collection at the Cariri Regional University—URCA (collection numbers LZ-URCA 1328 and LZ-URCA 1329). This work was approved by the Animal Ethics Committee of the Universidade Regional do Cariri—URCA under the Reference no.: 04/2012. 

### 2.2. Preparation of the Oil from *Phrynops geoffroanus* (OPG)

The fixed oils present in body fat located in the ventral regions of the turtles were extracted with hexane (60°C) for 6 h in a Soxhlet apparatus. The hexane was subsequently decanted and filtered and the solvent was removed by heating in a water bath at 70°C for 2 h; the extracted oil was subsequently stored in a freezer for future analysis. 

### 2.3. Determination of the Fatty Acids

The fatty acids in the OPG were analyzed indirectly by identifying their corresponding methyl esters. The extracted oil (0.2 g) was saponified by refluxing for 30 min. in a solution of potassium hydroxide and methanol, following the methodology described by Hartman and Lago [19]. The pH of the extract was adjusted, and the free fatty acids were methylated by acid catalysis to generate their methyl esters. 

### 2.4. Analysis of OPG by Gas Chromatography Coupled to a Mass Spectrometer (GC/MS)

The analysis of volatile constituents was carried out in a Hewlett-Packard GC/MS, model 5971, using the nonpolar DB-1 fused silica capillary column (30 m × 0.25 mm i.d., 0.25 *μ*m film), eluted with helium gas at 8 mL/min with split mode. Injector and detector temperatures were set to 250°C and 200°C, respectively. The column temperatures was programmed from 35°C to 180°C at 4°C/min and then from 180°C to 250°C at 10°C/min. Mass spectra were recorded from 30 to 450 *m/z*, with an electron beam energy of 70 eV. The individual components were identified by computer MS library searches, using retention indices as a preselection routine and visual inspection of the spectra from the literature for confirmation [20], as well as by visually comparing standard fragmentation to that reported in the literature [21, 22].

### 2.5. Microorganisms

Experiments were undertaken using clinical isolates of the bacteria *Escherichia coli* (*EC*27), *Staphylococcus aureus* 358 (SA358), and *Pseudomonas aeruginosa (PA*22). The strains *Escherichia coli *ATCC-10536, *Staphylococcus aureus* ATCC-25923, *Pseudomonas aeruginosa* ATCC-15442, and *Klebsiella pneumoniae* ATCC-4362 were used as positive controls. Isolates of* Candida albicans* ICB12 and *Candida krusei* 6258 were used to evaluate antifungal activity, as well as in the modulation tests [23]. All of the lineages were maintained in* heart infusion agar slants *(HIA, Difco). The cells were cultivated during the night before the trials at 37°C in* a Brain Heart Infusion medium *(BHI, Difco).

### 2.6. Drugs

The antibiotics gentamicin, amikacin, and neomycin were obtained from Sigma Chemical Corp., St. Louis, MO, USA. The antifungal drugs used were amphotericin B (Sigma Co., St. Louis, USA), mebendazol (Lasa—Pharmaceutical Industries LTDA., Brazil), nystatin (Laboratório Teuto Brasileiro S/A, Brazil), and metronidazole benzoate (Prati, Donaduzzi and Cia LTDA., Brazil). All of these compounds were dissolved in sterile water before use.

### 2.7. Tests of Drug Susceptibility

A test solution of OPG was prepared using 20 mg of the oil dissolved in 1 mL of dimethyl sulfoxide (DMSO) (Merck, Darmstadt, Germany), generating an initial concentration of 20 mg/mL. This solution was subsequently diluted to 1024 *μ*g/mL with sterile water. The minimum inhibitory concentrations (MICs) of the oil were determined in BHI using microdilution series with suspensions of 10^5^ CFU/mL and drug concentrations of 1024 *μ*g/mL to 1 *μ*g/mL (double dilution series) [24]. The MIC was defined as the lowest concentration of a compound that inhibited microbial growth. To investigate the potential of the oil as a modulator of antibacterial and antifungal drug activities, we determined the antibacterial (128 *μ*g/mL) and antifungal (16 *μ*g/mL) MICs in subinhibitory concentrations; the plates were incubated for 24 hours at 37°C.

## 3. Results 

The body fat of *Phrynops geoffroanus* was found to be composed of 84.63% and 13.38% of unsaturated and saturated methyl esters, respectively (Table 1).

OPG alone did not demonstrate any clinically relevant antibacterial activity, with a MIC ≥ 1024 *μ*g/mL for all of the bacterial strains tested—indicating that the body fat of this turtle is inefficient when used alone in treating bacterial infections. When tested against fungal strains, the OPG demonstrated a MIC ≥ 1024 *μ*g/mL against *Candida albicans* ICB12 and MIC 128 *μ*g/mL against *C. krusei* 6258, thus demonstrating clinically relevant antifungal activity against infections caused by the latter fungus. The OPG was also tested for possible antibacterial and antifungal activity when combined with commonly used antibiotics or antifungal drugs. Tests to confirm possible synergisms between OPG and aminoglycosides (Table 2) were negative against multi-resistant strains of *E. coli *(EC27) and *Staphylococcus aureus *(SA358). A synergistic effect was noted, however, against *Pseudomonas aeruginosa* (PA22) when OPG was combined with gentamicin. Tests of the modulation of antifungal drugs when associated with OPG, likewise do not demonstrate positive effects against *Candida albicans* ICB12 or *Candida krusei* 6258 (both having MIC ≥ 1024 *μ*g/mL).

## 4. Discussion

The antibacterial and antifungal properties of several fatty acids (FAs) were reported [25, 26] and according to Zheng et al. [27], the mechanism of the antimicrobial activity is related to the action of the unsaturated fatty acids affecting the synthesis of the endogenous microbial fatty acids. The OPG is composed mainly of unsaturated fatty acids (84,63%), being the main component the palmitoleic and oleic acids (58,39% and 15,7%, respectively). The high concentration of unsaturated FAs is different when compared with the FAs composition observed in the work of Scarlato and Gaspar [28], but similar to the work of Gaspar and Silva [29], both studying different parts of *Podocnemis expansa*. 

The OPG when assayed alone against bacterial and fungal strains demonstrated an clinically nonrelevant antimicrobial activity against all strains, except against *C. krusei*, (MIC = 128 *μ*g/mL). When the OPG was associated with antibiotics and antifungal drugs, was observed a synergistic effect when associated the OPG with the gentamicin against the strain *Pseudomonas aeruginosa* (PA 22) (Table 2). In studies with the body fat of the lizard *Tupinambis merianae*, Ferreira et al. [30] demonstrated that besides the usage of this body fat in the folk medicine against infectious diseases, this product demonstrated non-antimicrobial activity when used alone or associated with antibiotics. The body fat of *T. merianae* presented 57 and 43% of unsaturated and saturated FAs respectively. Possibly, the antimicrobial and modulatory activity demonstrated by OPG can be due to the high percent of unsaturated FAs detected in this work.

Our results demonstrated that the body fat of *P. geoffroanus* has antimicrobial and modulatory activities, this work being the first report about a biological activity by this product, validating its usage by the folk medicine and indicating a clear relationship between the percent of unsaturated fatty acids and this antimicrobial and modulatory activities. 

## 5. Conclusion

The results of our work validate the antimicrobial and modulatory activities of OPG and possibly, of other body fats with high level of unsaturated fatty acids. Due this fact, we recommend more studies to evaluate the use of the body fat of *P. geoffroanus* against other diseases informed by ethnopharmacological surveys.

## Figures and Tables

**Table 1:** GC/MS characterization of the methyl esters of the fatty acids in oil from the body fat of *P. geoffroanus*.

Name	T_*r*_ (min)^a^	(%)
Perlagonic acid	11,43	2,04
Pentadecylic acid	19,09	3,68
Palmitoleic acid	22,31	58,39
Caprylic acid	23,98	0,84
Linoleic acid	24,63	4,50
Linolenic acid	24,72	2,28
Oleic acid	24,83	15,70
Erucic acid	24,90	3,76
Palmitic acid	25,13	6,82
Saturated esters	—	13,38
Unsaturated esters	—	84,63
Total	—	98,01

^a^Retention time.

**Table 2:** MIC values (*µ*g/mL) of aminoglycosides against *Escherichia coli* 27, *Staphylococcus aureus* 358, and *Pseudomonas aeruginosa* 22 in the absence and presence of 128 *µ*g/mL of oil derived from the body fat of *P. geoffroanus*.

Antibiotics	EC 27		SA 358		PA 22	
	MIC	MIC Combined	MIC	MIC Combined	MIC	MIC Combined
Amikacin	4,9	4,9	19,5	19,5	312,5	312,5
Neomycin	4,9	4,9	19,5	19,5	312,5	312,5
Gentamicin	2,44	2,44	9,8	9,8	39,1	9,8
